# Biosensing Using Microring Resonator Interferograms

**DOI:** 10.3390/s140101184

**Published:** 2014-01-10

**Authors:** Shih-Hsiang Hsu, Yung-Chia Yang, Yu-Hou Su, Sheng-Min Wang, Shih-An Huang, Ching-Yu Lin

**Affiliations:** 1 Department of Electronic Engineering, National Taiwan University of Science and Technology, No.43, Sec. 4, Keelung Rd., Taipei 10607, Taiwan; E-Mails: d9902308@mail.ntust.edu.tw (Y.-C.Y.); s90045555@yahoo.com.tw (Y.-H.S.); s960318@gmail.com (S.-M.W.); forsam10@gmail.com (S.-A.H.); 2 School of Medical Laboratory Science and Biotechnology, College of Medical Science and Technology, Taipei Medical University, 250 Wu-Hsing Street, Taipei 11031, Taiwan

**Keywords:** interferometry, biophotonics, microring resonator, silicon wire, multimode interference, birefringence

## Abstract

Optical low-coherence interferometry (OLCI) takes advantage of the variation in refractive index in silicon-wire microring resonator (MRR) effective lengths to perform glucose biosensing using MRR interferograms. The MRR quality factor (*Q)*, proportional to the effective length, could be improved using the silicon-wire propagation loss and coupling ratio from the MRR coupler. Our study showed that multimode interference (MMI) performed well in broad band response, but the splitting ratio drifted to 75/25 due to the stress issue. The glucose sensing sensitivity demonstrated 0.00279 meter per refractive-index-unit (RIU) with a *Q* factor of ∼30,000 under transverse electric polarization. The 1,310 nm DFB laser was built in the OLCI system as the optical ruler achieving 655 nm characterization accuracy. The lowest sensing limitation was therefore 2 × 10^−4^ RIU. Moreover, the MRR effective length from the glucose sensitivity could be utilized to experimentally demonstrate the silicon wire effective refractive index with a width of 0.45 μm and height of 0.26 μm.

## 1. Introduction

Optical waveguide sensors have attracted considerable attention due to their immunity to electromagnetic interference, good compactness and robustness and high compatibility with fiber networks, while exhibiting shorter response times and higher sensitivities [1]. In just a few years researchers have proposed using microring resonator (MRR) for label-free biosensing applications [2–5]. The high quality factor (*Q*) caused by the strong electric field enhancement within the ring makes the MRR a good candidate for biomolecule detection under low analyte concentration conditions. A MRR consists of a ring, bus waveguide and optical coupler, that utilize a directional coupler or multimode interference (MMI) for coupling. The operating function allows for light to evanescently couple into the ring through the optical coupler when a phase match occurs between the incident light and resonator whispering galley mode. When resonance forms the wavelength shift can be utilized to quantify target molecule biorecognition. Therefore, most MRRs use the resonating wavelength shift induced by the cladding layer refractive index change in biosensing applications.

The interference fringe patterns (or interferograms) from the Mach-Zehnder (MZ) interferometer will represent the optical path difference in two MZ arms, in which variation causes the fringe position shift [6]. In biosensing approaches there are two main technologies, namely optical phase changes controlled by a high coherence source [7] and interferograms formed by optical low coherence [8–11]. In the typical optical intensity interference with a high coherence source, the accuracy is easily degraded by the output light power fluctuation and interferometric signal periodic nature. Interferograms from low coherence are relatively stable and the resolution limit can be enhanced two- fold in cascaded MZ interferometers [9].

Here, we propose an optical low coherence interferometry (OLCI) system including a fiber-optic MZ interferometer and a continuous-wave broad-band source used to characterize the high *Q* MRR on one MZ arm using the optical ruler for resolution enhancement. Glucose is utilized as an analyte and sensed using spatially-localized interference fringe patterns or interferograms for refractive index biosensors. The typical low-coherence interferogram sensor did not include the optical ruler, which is crucial for test accuracy. Furthermore, the proposed optical ruler in the OLCI was not located in the same optical path as the device under test (DUT), which meant that the wavelength utilized for an optical ruler will not be limited by the DUT wavelength selection. Since the optical paths between the optical ruler and DUT are different, a high coherent short wavelength with free space light tracking could be applied to the optical ruler for higher sensitivity. Refractive index sensors usually use the optical power or wavelength related for sensing. The former sensitivity is easily affected by the light power fluctuation and the sensitivity of the latter is limited by the availability of a costly high resolution optical spectrum analyzer (OSA). Different glucose concentrations could be derived using silicon-wire waveguide birefringence from relative interferogram movement distances.

## 2. Design and Theory

**Figure 1.**
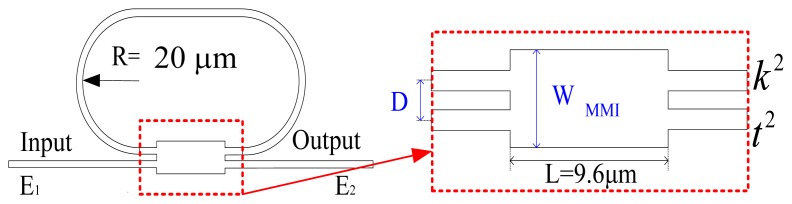
A ring resonator using a MMI coupler.

Because interferograms are demonstrated using low coherence sources, all elements in the OLCI system require broad band response. A schematic of the MMI-coupled MRR and MMI structure is illustrated in Figure 1. A MMI coupler is used for MRR coupling, shown in the inset of Figure 1. The power transmission of MRR in the bus waveguide can be expressed as [12]:

(1) $${\left|T\right|}^{2}={a_{\text{MMI}}}^{2}\left[\frac{\alpha^{2}-2\alpha t\cos\theta +t^{2}}{1-2\alpha t\cos\theta +\alpha^{2}t^{2}}\right]$$

where *t^2^* is the coupler power self-coupling coefficient, *α^2^* is the power loss factor which includes both the ring loss and the coupler loss, *α^2^* =*α*_MMI_*^2^*·*α*_ring_*^2^*, and *θ* is the round trip phase accumulation.

The quality factor *Q* of a MRR can also be derived and demonstrated as follows [12]:

(2) $$Q\equiv \frac{\lambda }{\Delta \lambda_{\text{FWHM}}}≅\frac{\pi n_{g}d}{\lambda }\frac{\sqrt{\alpha t}}{1-\alpha t}$$

where *λ* is the wavelength in vacuum and Δ*λ_FWHM_* is the full resonant wavelength width at half maximum. *n_g_* is the ring waveguide group index. *d* is the perimeter of the ring resonator. *k^2^* is the coupler power cross-coupling coefficient and equal to 1−*t^2^*, as shown in the inset of Figure 1.

The MMI, which is insensitive to the wavelength and has large processing tolerance, is used as the MRR coupling function. In this research we used the paired interference self-imaging MMI for equal splitting ratios, which requires the MMI length to be half the beat length [13]. The finite difference time-domain (FDTD), a direct solution of Maxwell's curl equations generated using the RSoft Fullwave tool, was utilized to simulate and predict the modal performance for silicon-wire waveguides based on the MMI coupler. The MMI for both the transverse electric (TE) and transverse magnetic (TM) polarizations was designed to have a length (L) of 9.6 μm, width (W) of 3 μm and input/output waveguide distance (D) of 1 μm when the silicon-wire waveguide has a width of 0.45 μm and height of 0.26 μm.

**Figure 2.**
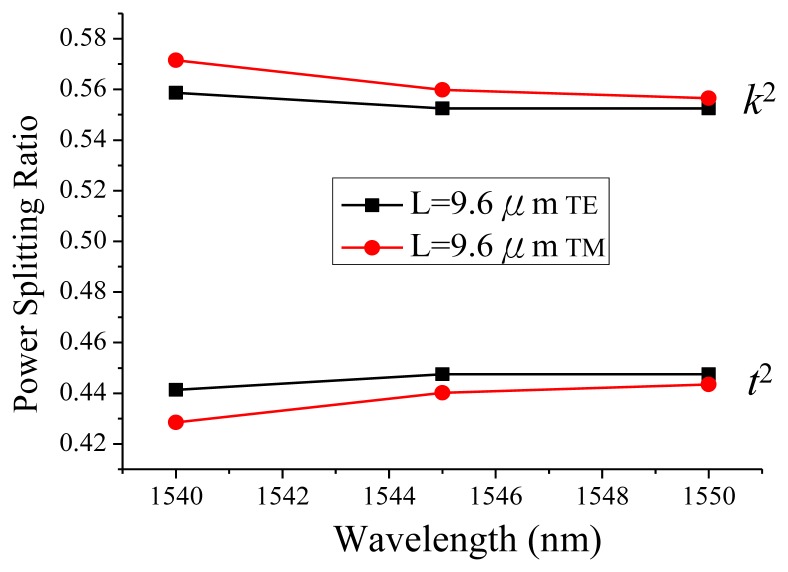
FDTD simulated MMI coupler performance.

The FDTD is a 3D simulation of the whole MMI structure. The boundary conditions for a cross section with the same mesh size of 0.035 μm were −3 to 4 μm and −0.77 to 0.98 μm for the horizontal and vertical directions, respectively. A simulated mode from the current silicon-wire waveguide structure was taken as the launching field instead of the default selection and the simulation time was about 45 min. The power splitting ratio was calculated as 0.56 and 0.44 for *k*^2^ and *t*^2^, respectively. The optical power ratio difference between TE and TM polarizations was less than 0.01, as shown in Figure 2.

The effective length *L_eff_* of the MRR could be written as follows [14,15]:

(3) $$L_{\text{eff}}\equiv \frac{Q\lambda }{2\pi n_{\text{eff}}}$$

where *n_eff_* is the effective index of the ring resonator waveguide.

The OLCI phase is represented by the interferograms and composed of the light wave propagation constant and interferometer arm lengths. Since a MRR with a narrow line width could achieve higher *Q* and longer effective length, the optical path difference between two interferometer arms is more easily detected by the cladding layer refractive index change. This relationship can be given using the following when the *L_eff_* from MRR is fixed and invariant with the environmental change:

(4) $$\begin{array}{ccc} \Delta \phi =k[n\Delta L_{\text{eff}}+\Delta nL_{\text{eff}}] & \underset{\to }{\Delta L_{\text{eff}}=0} & \Delta \phi =k\Delta nL_{\text{eff}}=k\Delta L_{MD} \end{array}$$

where Δ*n* is the refractive index variation only caused by the cladding layer and *k* is the wave number. Δ*L_MD_* is the relative moving distance between interferograms from OLCI.

When the relative moving distance Δ*L_MD_* could be affected by the various refractive indices Δ*n*, the slope is represented by the MRR effective length *L_eff_*. The longer the MRR effective length is the higher the sensing limit that can be achieved. On the other hand, the silicon-wire waveguide effective refraction index in Equation (3) is related to the MRR effective length, which is the curve slope from Δ*L_MD_* and Δ*n* in Equation (4). If polarization is applied to the MRR for different glucose sensing levels, the silicon wire effective index of refraction and birefringence can be derived from MRR interferograms.

## 3. Experiments and Discussions

**Figure 3.**
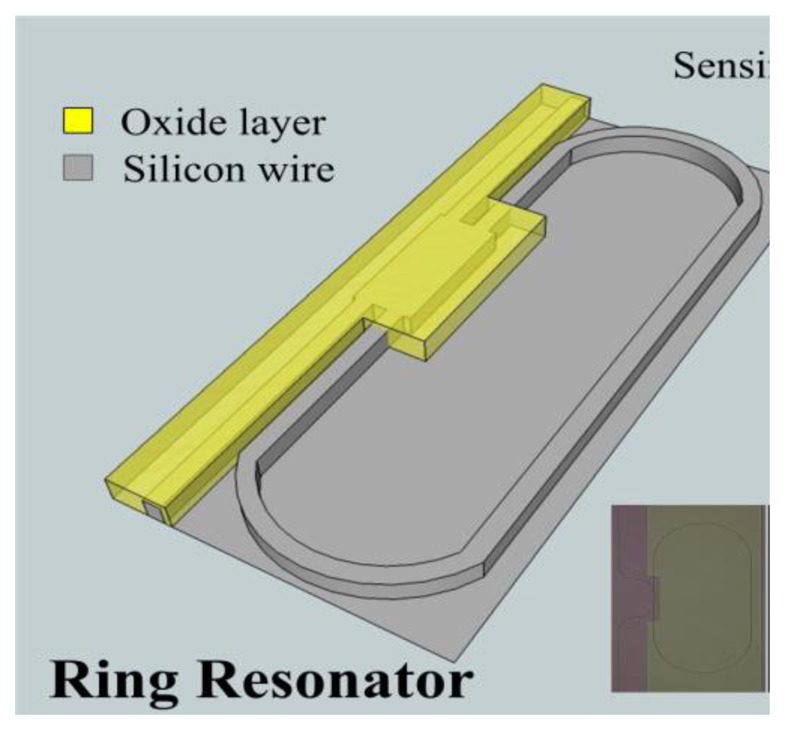
Schematic drawing for the optical ring biosensors (Insets: the optical image for a biosensor sensing part and SEM picture for silicon wire waveguide).

The top silicon layer and box layer of the silicon-on-insulator (SOI) wafer were 0.26 μm and 3 μm, respectively. Following RCA cleaning the SOI wafer was spin coated with 800-nm positive photoresist. I-line stepper lithography was implemented to pattern the silicon wire waveguides with reactive ion etcher utilized to fabricate the MMI coupled MRR on the silicon-wire rib waveguide with the 0.45 μm width and 0.26 μm height. At room temperature three main gas mixtures, Cl_2_, HBr, and O_2_ were used to obtain anisotropic silicon structures. In this processing mixture, Cl_2_ could react with silicon to form a polymer for sidewall passivation and provide etch anisotropy. The MMI and input/output waveguide were next covered by a 300 nm thick silicon dioxide with the ring perimeter left open for analyte sensing, as shown in Figure 3. The MMI coupler MRR image was taken using an optical microscope camera, as illustrated in the inset of Figure 3. The scanning electronic microscope (SEM) for silicon-wire waveguides is also shown in the inset of Figure 3.

To understand the coupling coefficient for the MMI coupled MRR, an additional MMI with the same structure was intentionally allocated nearby for optical power splitting function analysis. The MMI is a 2 × 2 coupler and *t^2^* and *k^2^* are the self- and cross-coupling coefficients, respectively. Therefore, *k^2^* represents the optical power coupled to MRR.

**Figure 4.**
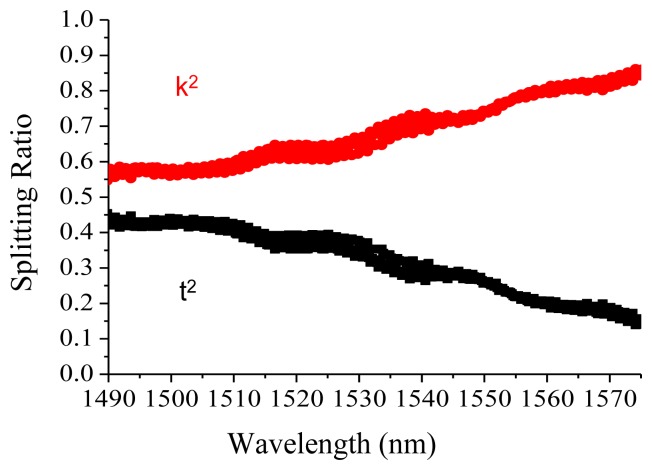
The MMI splitting ratio under TM polarization.

**Figure 5.**
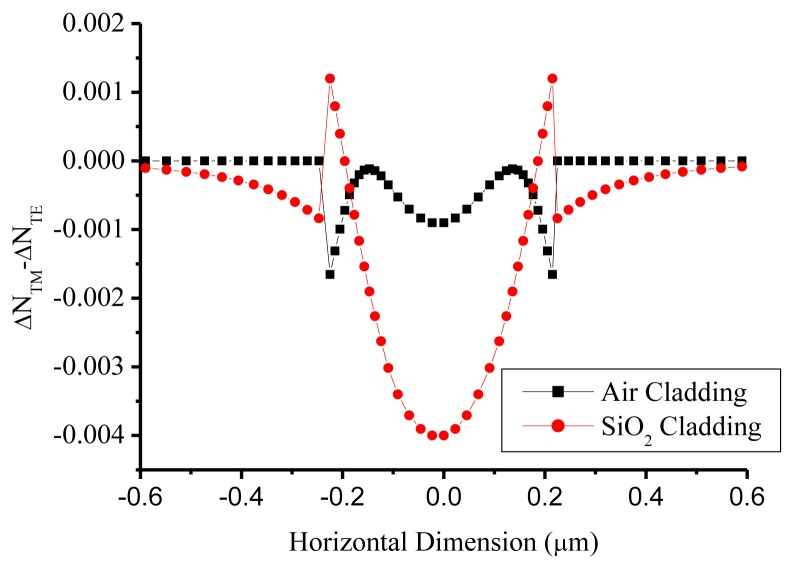
Simulated birefringence caused by the stress.

The MMI for the TM polarization is characterized in Figure 4 to have power ratios as 0.75 and 0.25 at 1,548.58 nm wavelength for *k^2^* and *t*^2^, respectively, different from the theoretical calculation of 0.56/0.44. After the stress effect caused by the single 300 nm silicon dioxide cladding layer was considered, the calculated phase birefringence, which was proportional to stress effect [16] and executed by the Thermo Scientific C2V stress module, demonstrated non-uniform distribution in the silicon-wire waveguide, as shown in Figure 5. The air cladding was simulated for the birefringence comparison. The biggest birefringence occurred at the center of the silicon wire waveguide, where the most optical power was located, indicating that the MMI splitting ratio was significantly affected. The experimental splitting ratios of 0.75 and 0.25 were matched to the previous publication [12].

The bus waveguide and the coupler region were covered with plasma-enhanced chemical vapor deposition (PECVD) oxide. Our preliminary data showed the propagation loss difference between the air and PECVD oxide claddings on the silicon wire waveguides was around 0.1–0.2 dB/cm, which was matched to the previous publication [17]. In order to obtain the high *Q* for a long effective length, the MMI coupled MRR was studied with the lowest propagation loss and ring resonator perimeters. Our data showed that the silicon propagation loss was 5 dB/cm. The optical MMR perimeter was 3,368 μm with 20 μm bending radius.

**Figure 6.**
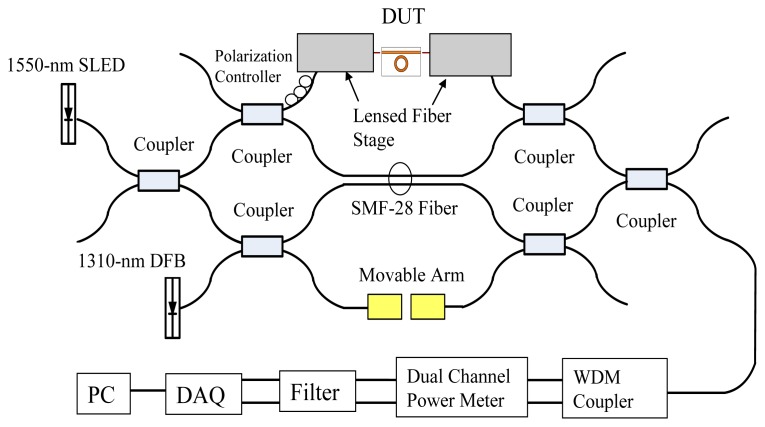
Characterization for the optical ring resonator.

**Figure 7.**
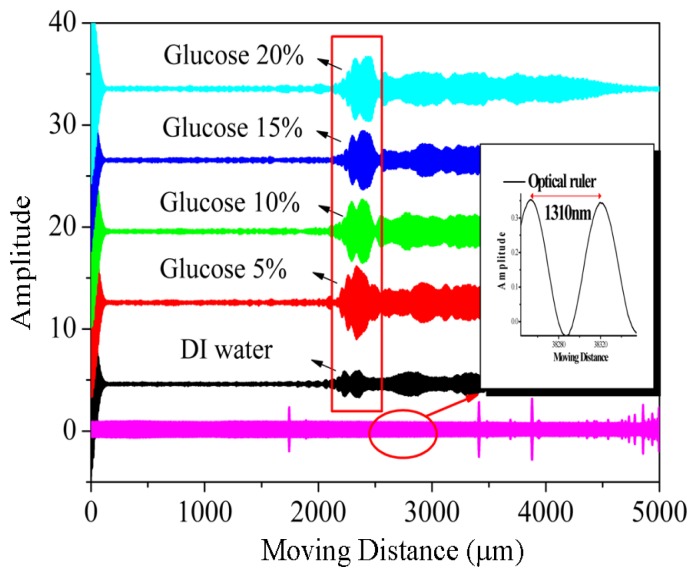
The interferograms from various glucose concentrations in TE polarization.

The OLCI included a fiber-optic MZ interferometer and a continuous-wave broadband source was utilized for characterization. The optical path difference between the two interferometer arms was changed by translating a motor driven stage, as shown in Figure 6. Therefore, a variable-delay interferometer could be illustrated using a spatially-localized interference fringe pattern or interferogram. The experimental setup separated TE and TM polarizations using a polarization controller. A super-luminescent diode (SLD), with a 3 dB spectral width of 60 nm and maximum output at 1,552 nm wavelength, involved with a 1,310 nm wavelength distributed feedback (DFB) laser as the optical ruler and propagated through OLCI where the DUT silicon-wire waveguide was installed in one of three arms. The collimators were placed onto a movable arm located on another MZ stage and controlled by a stepper motor. The optical power was analyzed using a dual channel power meter and the electrical filter and data acquisition (DAQ) card were executed for the noise filtering and analog/digital conversion functions. The relative collimator movement distance represents the interferogram phase difference and can derive the MRR effective length from Equation (4). A SMF-28 fiber functioned as the reference and demonstrated the first interferograms for every scan movement in Figure 7. To distinguish interferograms from the optical ruler, deionized water (DI) water and glucose concentrations of 5%, 10%, 15% and 20% in TE polarization, each interferogram intensity was intentionally shifted away from each other for around 10 arbitrary unit (A.U.) separation, as shown in Figure 7.

**Figure 8.**
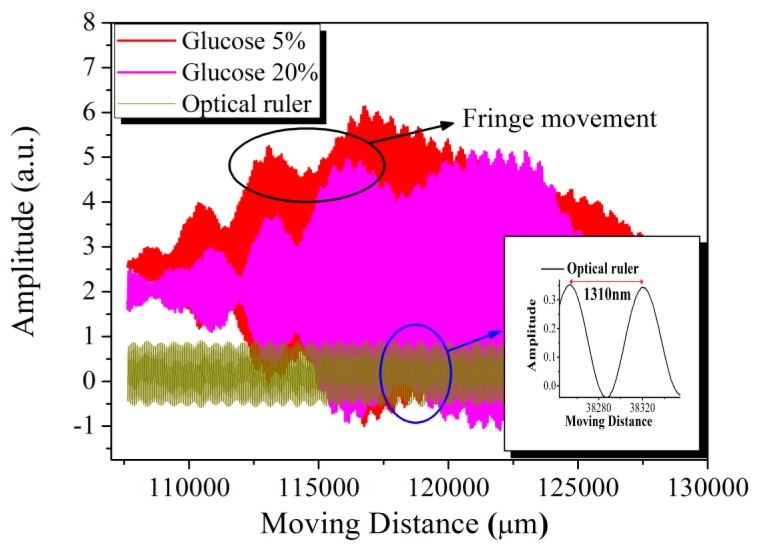
The relative movement distance between interferograms.

In Figure 7, the interferograms from glucose concentrations of 5% and 20% were taken to illustrate the relative movement distance. Curve fitting was applied on the selected peaks and the fringe movement was gauged from the relative distance between the full width at half maximum (FWHM) centers for each fitted curve, as shown in Figure 8.

**Figure 9.**
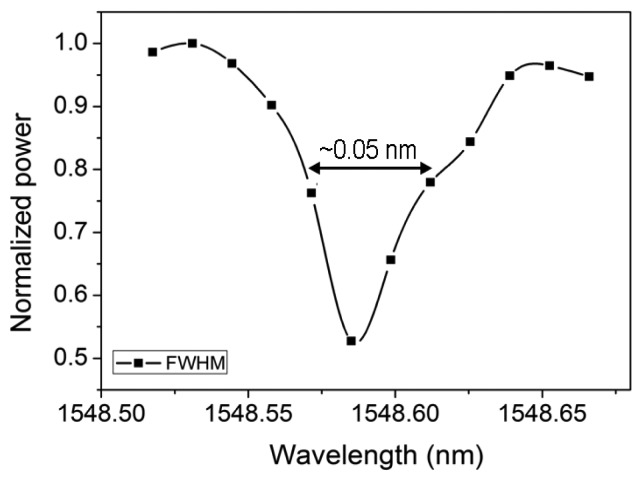
The FWHM of the MRR resonance from thermal DFB wavelength tuning.

**Figure 10.**
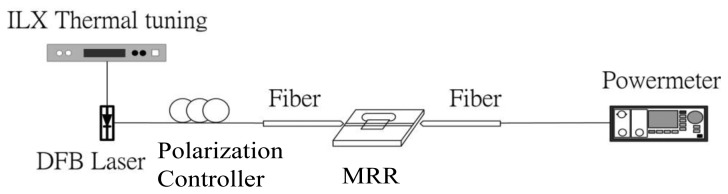
The test setup for MMR *Q* factor characterization.

Due to the OSA resolution 0.1 nm limitation, the MRR resonance could not be distinguished well enough for the line width characterization. After considering the MMI coupler power cross-coupling coefficient as 0.75, the *Q* factor was then derived as ∼30,000 from the waveguide propagation loss 5 dB/cm for *α*, and MMI self-coupling coefficient 0.25 for *t^2^* after *n_g_* and *d* were inserted as 4 [18] and 3,368 μm, respectively, from Equation (2). Furthermore, the DFB laser with the most stable 0.1 °C thermal tuning was utilized for testing, the FWHM demonstrated around 0.05 nm and the *Q* factor was ∼30,000, as shown in Figure 9 and the testing setup is shown in Figure 10.

**Figure 11.**
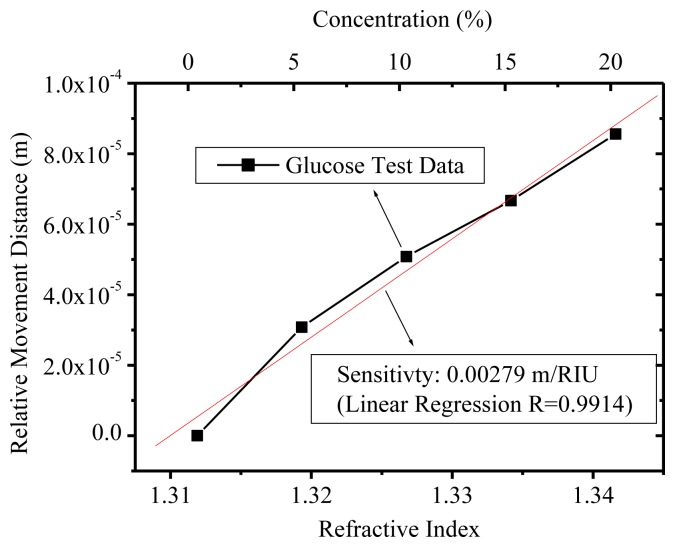
The sensitivity from the refractive index and relative movement distance in TE polarization.

**Figure 12.**
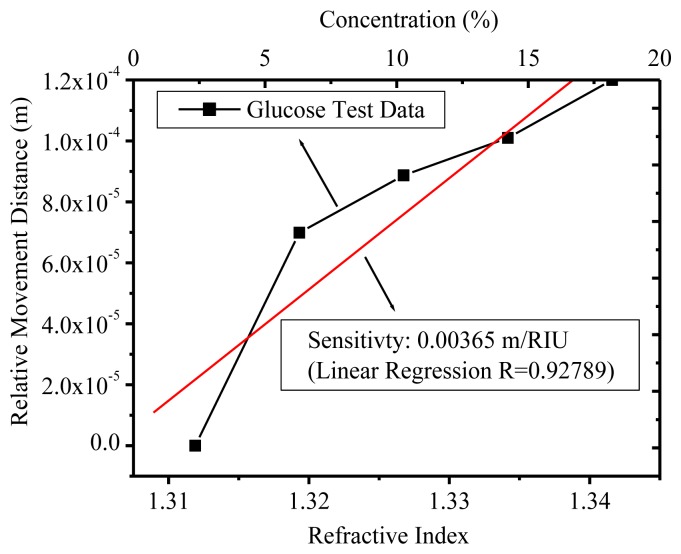
The sensitivity from the refractive index and relative movement distance in TM polarization.

After the quality factor *Q* for an optical MRR was experimentally characterized as ∼30,000 at 1,548.58-nm wavelength, various glucose concentrations were prepared to increase from 5%, 10%, 15%, and 20%. The interferograms and their relative movement distances for TE polarization are illustrated in Figure 7. The fiber based OCLI system was sensitive to the polarization and the relative movement distance resolution was well controlled with 655 nm (= 1,310/2 nm) accuracy. We conclude that the sensing limitation was 2 × 10^−4^ (= 655 nm/0.00279 m) refractive index unit (RIU) from Equation (4). The glucose concentration could also be calculated as the refractive index [19] shown in Figure 11. The sensitivity was demonstrated as 0.00279 m/RIU after the linear fitting, which was *L_eff_* in Equation (4). From Equation (3)*n_TE_* could be derived as 2.65. The TM polarization was characterized, as shown in Figure 12, and its sensitivity was shown as 0.00365 m/RIU, which is more sensitive than the TE mode.

The TE curve is better than TM for linear regression and it may come from the rough top surface of the silicon wire waveguide due to the 800 nm photoresistor surviving failures in silicon core layer over-etch during RIE processing.

## 4. Conclusions

The proposed biosensing element is based on the SOI MRR effective length and takes advantage of its refractive index variation from interferograms for analyte detection. The waveguide effective index of refraction can also be derived from the MRR effective length. Our data demonstrated that the sensitivity was 0.00279 m/RIU and the sensing limitation achieved 2 × 10^−4^ with the optical ruler from 1,310 nm DFB laser. The sensitivity and detection limit could be further improved using MMI stress compensation.
